# Emergent geometries and nonlinear-wave dynamics in photon fluids

**DOI:** 10.1038/srep23282

**Published:** 2016-03-22

**Authors:** F. Marino, C. Maitland, D. Vocke, A. Ortolan, D. Faccio

**Affiliations:** 1 CNR-Istituto Nazionale di Ottica, L.go E. Fermi 6, I-50125 Firenze, Italy; 2 INFN, Sez. di Firenze, Via Sansone 1, I-50019 Sesto Fiorentino (FI), Italy; 3 School of Engineering and Physical Sciences, SUPA, Institute of Photonics and Quantum Sciences Heriot-Watt University, Edinburgh EH14 4AS, UK; 4 INFN, Laboratori Nazionali di Legnaro, Viale dell’ Universita 2, I-35020 Legnaro (PD), Italy

## Abstract

Nonlinear waves in defocusing media are investigated in the framework of the hydrodynamic description of light as a photon fluid. The observations are interpreted in terms of an emergent curved spacetime generated by the waves themselves, which fully determines their dynamics. The spacetime geometry emerges naturally as a result of the nonlinear interaction between the waves and the self-induced background flow. In particular, as observed in real fluids, different points of the wave profile propagate at different velocities leading to the self-steepening of the wave front and to the formation of a shock. This phenomenon can be associated to a curvature singularity of the emergent metric. Our analysis offers an alternative insight into the problem of shock formation and provides a demonstration of an analogue gravity model that goes beyond the kinematic level.

Geometry plays a fundamental role in the description of disparate phenomena across different areas of physics, ranging from continuous mechanics and nonlinear dynamics to electromagnetism and high-energy physics. The paradigmatic example is General Relativity (GR) where, far from being merely a convenient representation, the language of differential geometry allows us to capture the essence of gravitational force. A recent active field of research in which geometrical concepts naturally comes into play is analogue gravity (for a review, see^1^). The general idea is that the propagation of a scalar field on a curved spacetime can be reproduced in condensed-matter systems by studying the evolution of elementary excitations on top of a suitable background configuration. For instance, sound waves in flowing fluids propagate in an effective Lorentzian geometry (acoustic metric) which is determined by the physical properties of the flow^2^,^3^,^4^. Hence, by properly choosing the background flow, it is possible to mimic black holes and several aspects of quantum field theory in curved spacetime.

These studies are generally based on perturbative schemes, i.e. they consider the propagation of linearized fluctuations over a given background solution of the full nonlinear problem. So, while the curved spacetime determines the propagation of waves, it is not affected by the waves propagating on it. As such, the analoguey works only at the kinematical level: the geometrical description holds only for the fluctuations, whose evolution is governed by linear wave equations, while the background couples with the usual flat metric.

A new perspective was opened by Goulart in ref. 5. Under certain conditions, the nonlinear dynamics of a scalar field can be described in terms of an emergent spacetime geometry, which is generated by the field itself and determines its propagation. This result extends the analogue gravity approach as the emergent metric now includes the whole dynamics of the system (i.e. even the evolution of the background) and is not restricted to linearized fluctuations. Remarkably, a similar situation occurs in GR: the metric on which a given scalar field propagates is modified by the scalar field itself, thus being a *dynamic* structure. In this framework, the wave propagation becomes a intrinsically nonlinear problem due to the coupling (back-reaction) between the wave and the metric^6^.

We note that the emergent metric in nonlinear models does not depend on the scalar field via Einstein’s equations and thus they cannot be directly considered as models for Einstein gravity. Nevertheless, they encode in a geometric framework the dynamical interplay between the scalar field and the metric and so they could provide valuable insights into relevant dynamical features present also in GR such as back-reaction effects at a full nonlinear level^7^.

Compressible, non viscous fluids appear as the ideal candidates for this kind of investigation and in principle, can push the analoguey further. A first connection between nonlinear hydrodynamics and differential geometry dates back to the Riemann’s work on discontinuous flows in 1860^8^. Since then, there have been numerous contributions, who laid the foundations of the modern mathematical theory of shock waves^9^,^10^. In the linear regime, hydrodynamic systems are the prototype model for studies on acoustic black holes^1^, Hawking radiation^2^,^3^,^4^ and superradiance^11^,^12^,^13^,^14^. Extending to the nonlinear regime, we find that causality is still governed by an acoustic metric^15^,^16^ and the evolution of nonlinear perturbations can be described through the analogue gravity formalism^17^. Acoustic geometries which dynamically depend on the fluid variables appear also in the context of black-hole accretion models^18^,^19^ and spherically symmetric outflows of nuclear matter^20^. Even more importantly, a precise correspondence exists between the gravitational field equations and suitably constrained nonlinear flows^21^. Therefore such systems could be exploited to mimic some aspects of black holes dynamics and spacetime singularities^22^.

As an alternative to actual flowing fluids, light beams propagating in defocusing optical media are promising models for analogue gravity experiments^23^,^24^,^25^,^26^,^27^. The optical field dynamics can be described as a fluid of interacting photons^28^,^29^ on which linear perturbations, i.e. sound waves, experience an effective curved spacetime determined by the field intensity and phase pattern.

Here, we report an experimental study of the dynamics of *nonlinear* acoustic disturbances in such photon fluids and we provide a geometrical interpretation of the results. We show that nonlinear density waves induce a background flow which, in turn, strongly modifies their propagation. In analoguey with GR, this self-interaction is interpreted in terms of an effective curved geometry generated by the wave itself. In particular, as observed in compressible, non viscous fluids, different points of the wave profile propagate at different velocities leading to the self-steepening of the wave front and the subsequent formation of a shock. This phenomenon is associated to a curvature singularity of the emergent metric.

Let us remark that this work is by no means an attempt to realize a perfect analogue of a realistic gravitational theory. Our backreaction, i.e. how the spacetime is modified by the waves propagating on it, is encoded in a set of nonlinear equations which have not the properties of Einstein’s equations. One of the crucial ingredients in GR is the invariance of the theory under diffeomorphisms. An aspect of such symmetry is the possibility of rewriting the equations of motion in a general covariant form, i.e. making them invariant in form under and arbitrary change of coordinates. Our system, and typically non-relativistic theories, do not possess such property. Nevertheless, our geometrical analoguey allows us to describe the self-steepening effect as as the result of the backreaction on the wave by its own effective metric, although such gravitational-like interaction manifestly differs from that of GR.

## Results

### Photon-fluid model

We consider the propagation of a monochromatic optical beam of wavelength *λ*, in a 1D defocusing medium. The slowly varying envelope of the optical field follows the Nonlinear Schrödinger Equation (NSE)


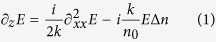


where *z* is the propagation direction, *k* = 2*πn*_0_/*λ* is the wave number and 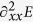, defined with respect to the transverse coordinate *x*, accounts for diffraction. The term Δ*n*(|*E*|^2^, *x*, *z*) describes the self-defocusing effect. In the case of thermo-optical media, the self-defocusing effect arises due to partial absorption of the light, which results in heating effects and, thus, to a decrease in the refractive index. Due to heat diffusion inside the medium, the nonlinearity can be can be highly nonlocal, i.e. the change in refractive index at any position depends not only on the local intensity, but also on surrounding field intensities^23^.

The nonlinear change of refractive index Δ*n* can be described in terms of a response function *R*(*x*, *z*) so that


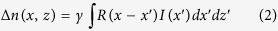


with *γ* being a normalisation factor and *I* = |*E*|^2^ the field intensity. In general, *R* depends on the nonlocal processes in the material and by the boundary conditions. In most cases the response is well described by an exponential decay *R*(*x*) = 1/(2*σ*)exp(−*x*/*σ*), where the critical length *σ* defines the degree of nonlocality^30^.

When nonlocal effects are negligible (*σ* ≈ 0), the nonlinear change of refractive index is Δ*n* = *n*_2_|*E*|^2^. In the Madelung formulation *E* = *ρ*^1/2^*e*^*iϕ*^, Eq. (1) can be recast into the continuity and Euler equation for a 1D-Bose-Einstein condensate


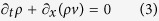



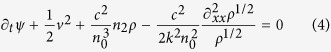


where the optical intensity *ρ* represents the fluid density, 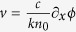 is the fluid velocity (*c* is the speed of light) and the propagation direction has been considered as a time coordinate 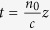. In Eq. (4), the optical nonlinearity 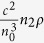 corresponds to the repulsive atomic interactions, while the last term is the analogue of the quantum pressure^31^. By applying the ∂_*x*_ operator to Eq. (4) and neglecting the quantum pressure term, we obtain


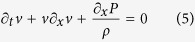


where we have defined the bulk pressure 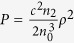.

Equations (3)–(5), 21, 21 are exactly the Navier-Stokes equations in a 1D compressible, non viscous fluid. While in the limit of small amplitudes, density perturbations behave as sound waves (i.e. linear fluctuations of the mean fluid flow), in the full nonlinear problem (3)–(5) a density wave cannot propagate forever. The wave profile steepens and eventually develops a discontinuity in a finite time^9^,^10^,^32^. The nonlinear term, *v*∂_*x*_*v*, determines the steepening rate of a wave. In real fluids this can be balanced by diffusion or viscosity, while in the photon fluids by the quantum pressure and nonlocality. If these terms are dominant over the nonlinearity, the self-steepening will be completely smoothed out. However, when these effects are sufficiently weak, a shock wave can form as the steepening wave approaches the discontinuity, where wave breaking occurs.

### Self-steepening regime

As discussed in the previous section, our system differs from an ideal compressible, non viscous fluid due to the presence of quantum pressure and nonlocal terms. Therefore, in order to clearly observe the self-steepening of density waves, it is necessary to operate in a regime where the above effects are negligible. We shall see that this regime is tightly related to the so-called phononic behaviour in the dispersion relation of small amplitude fluctuations. Let us briefly recall some results. The dispersion relation can be directly obtained by linearizing the nonlocal Eq. (1) around an homogeneous background, 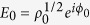 and by Fourier transforming^23^. We obtain


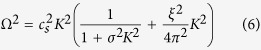


where *K* = 2*π*/Λ is the wavevector of the mode, Ω its angular frequency, 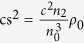 is the wave velocity and 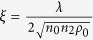. Using the definition of bulk pressure given in the previous section, we have that 
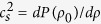
. Hence, *c*_*s*_ can be readily identified with the (constant) sound speed in the photon-fluid.

In the case of local media (*σ* = 0), Eq. (6) has the form of the Bogoliubov dispersion relation in a Bose gas, where *ξ* plays the role of healing length^33^,^34^. The characteristic length *ξ*, separates two different regimes. At low energies Λ ≫ *ξ*, we find the linear dispersion Ω ≈ *c*_*s*_*K*, typical of sound waves while at high energies the *K*^2^ quantum pressure contribution is dominant. In the case of nonlocal nonlinearities (*σ* > 0), an approximate linear dispersion is recovered by requiring both Λ ≫ *ξ*, and Λ ≫ *σ*. While these considerations can be hardly extended to the case of strongly nonlinear waves, they roughly identify the operational regime where the effects of the quantum pressure and nonlocal terms are less significant.

### Numerical simulations

The hydrodynamic representation of the NSE suggests that many features of acoustic waves can be investigated in the photon fluid using a pump-probe optical setup. Here a wide, flat-top beam (the pump) is injected into a defocusing thermo-optic medium together with a smaller Gaussian beam (the probe) tilted at an angle with respect to the pump. The superposition between the two beams generates an interference pattern, whose wavelength and amplitude are respectively determined by the tilt angle and relative intensities of the two beams. In the photon-fluid context, the pump beam corresponds to an homegeneous background fluid with constant density and zero flow velocity, while the interference pattern induces a density wave propagating on such background.

We numerically solved the NSE Eq. (1) with input conditions similar to the experiments described below and those shown in ref. 23. A pump pulse of 532 nm and beam diameter 1 cm is injected in a nonlinear defocusing medium with nonlinearity *n*_2_ = 8 × 10^−6^ cm^2^/W. A probe beam is overlapped at the input with a small 0.02 deg angle. For the sake of clarity, here we use a narrow probe, so that our the envelope of our initial condition has a width comparable to the wavelength.

**Figure 1:**
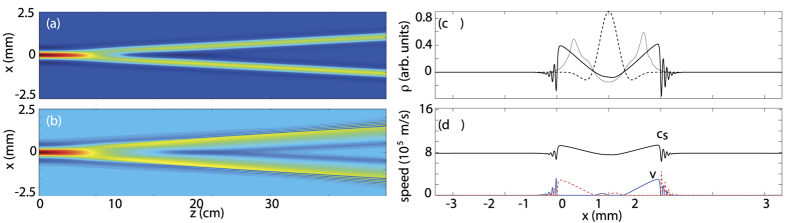
Numerical results: probe beam evolution in the presence of a flat-top pump beam obtained by numerical integration of Eq. (1). (**a**) (*σ* = 0) A weak probe beam (1% of the pump power, 0.02 deg input angle) splits into two beams corresponding to two Bogoliubov particles or density waves in the fluid propagating in opposite directions. (**b**) (*σ* = 0) At high probe intensities (same power as the pump) the density waves propagate nonlinearly and develop (supersonic) shock fronts that form ripples on the leading edges. (**c**) lineouts taken from (**b**): dashed line - input profile of the density wave. Thick solid line - density wave at z = 18 cm. Thin solid line - density wave at z = 18 cm for a nonlocal medium with nonlocal length *σ* = 110 *μ*m. (**d**) Sound velocity, *c*_*s*_, in the photon fluid (black line) and fluid flow speed (blue line - positive direction speed, dashed red line - negative direction speed).

For a small input amplitude (less than ~10% of the pump), the density wave is in the linear regime. The initial condition splits in two parts propagating in opposite directions. As previously discussed, the evolution after separation is governed by Bogoliubov dispersion relation^23^,^27^,^29^ which allows us to interpret these waves as Bogoliubov particles. At long-wavelengths, dispersive effects (quantum pressure) are negligible and density waves move at constant velocity (the sound speed) with no distorsion of their shape. An example of these dynamics is shown in Fig. 1(a). For higher probe powers the density waves enter into the nonlinear regime. After the separation, the density profiles steepen on the front edges and then breaks into higher frequency ripples, Fig. 1(b,c). This is due to the fact that the propagation velocity is now a function of the density, and therefore is different for different points in the profile. Since points of higher density propagate faster than points at lower densities, the profile increasingly steepens in the course of time. These results well reproduce the typical evolution of a density wave in ideal compressible, non viscous fluid up to the formation of flow discontinuities^32^.

As we will clarify later, the velocity of a point in the nonlinear wave profile is given by *v* ± *c*_*s*_, that are now both dependent on the density. Figure 1(d) shows lineouts of the sound speed *c*_*s*_ and background fluid velocity *v* corresponding to the wave profiles displayed in Fig. 1(c). As can be seen, both speeds are significantly modified due to high amplitude of the propagating waves. In particular, the density wave induces locally a nonzero flow velocity.

We recall that in linear acoustic analogues, the spacetime curvature is determined by the fluid velocity and the sound speed profiles (spatially homogeneous flows provide a flat spacetime). Here the background flow is induced by the wave itself. In other words, the initially flat spacetime geometry is curved by the waves, which are then in turn distorted by the spacetime metric. This suggests the possibility to achieve a geometrical description of the dynamics, as a kind of backreaction on the wave by its own effective metric.

### Experiments

**Figure 2:**
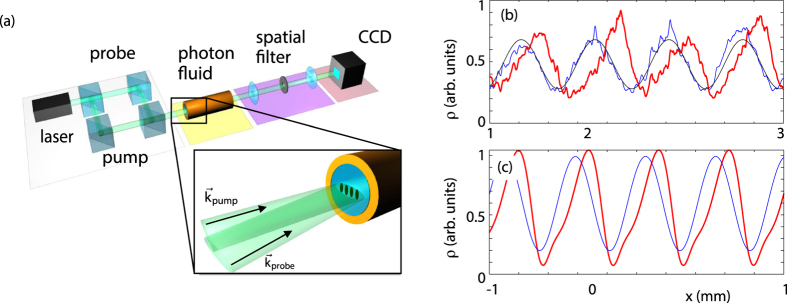
Experimental results. (**a**) experimental layout. (**b**) measured density wave profile after spatial filtering for low powers (I = 0.02 W/cm^2^, linear density wave propagation) and high power (I = 1 W/cm^2^, nonlinear propagation). (**c**) Numerical simulation under the same conditions of the experiment, including also the nonlocal medium response, with nonlocal length *σ* = 110 *μ*m.

The self-steepening of waves can be experimentally observed by using a similar setup to ref. 23 (see Fig. 2(a)). A 532 nm, CW laser beam is divided in two parts and then recombined forming a Mach-Zehnder interferometer setup. The more intense pump beam (1 W/cm^2^) forms the background fluid and the weaker probe beam in injected at a small angle so as to create, by interference, a density wave in the photon fluid. Both the pump and probe beams are focused into the sample using a cylindrical lens pair to create an elliptical beam with a very wide major axis diameter of 1 cm and a minor axis diameter of 260 *μ*m. The propagation dynamics occurs along the major axis and our system can be considered as one-dimensional. By controlling the relative angle and power of the two beams we created an interference pattern of the desired wavelength and modulation depth. This density wave is injected in a nonlinear sample, a 18 cm long cell filled with a methanol/graphene solution. Methanol has a negative thermo-optic coefficient but a low absorption in the visible. Nanometric graphene flakes (7 nm average size) are therefore dissolved in the medium in order to increase absorption. The absorbed energy is released in the form of heat, which in turn provides the defocusing nonlinearity via thermo-optic effect^23^. After the cell, the probe beam profile (i.e. the density wave) is isolated from the pump with a spatial filter, consisting of a pinhole in the far-field of the first lens of an imaging telescope and is then imaged onto a CCD camera.

As discussed in previous sections, a strong self-steepening effect can be observed if the quantum pressure and nonlocal terms play a marginal role, at least in the first stages of propagation. Therefore, the wavelength of the sound mode must be chosen such that Λ ≫ *ξ*, and Λ ≫ *σ*. The critical lenghts *ξ* and *σ* can be estimated by studying the propagation of small-amplitude fluctuations in our fluid for different pump powers (see measurements in ref. 23 and Methods for a description of the technique). For our experiment the nonlocal length *σ* over which the nonlinearity extends has been measured to be 110 *μ*m and, for the chosen pump power, the healing length is *ξ* = 85 *μ*m^23^. Here, the probe beam is incident at a 0.02 deg. angle, giving rise to a density wave with a wavelength of about 1 mm. The beam width has been chosen in order to observe several periods of the density wave.

Typical profiles of the photon fluid density waves are shown in Fig. 2(b). For low amplitude input the wave evolves as a linear perturbation of the background and preserves the initial sinusoidal shape (blue curve). Given its long wavelength, the density wave evolves as a sound wave with a constant sound speed determined by the background density. This regime and the complete dispersion curve have been exhaustively characterized in ref. 23. Let us remark that even such a small-amplitude wave would eventually end up to steepen and produce a shock. This is simply due to the fact that nonlinear effects are cumulative as the wave propagates. However, the smaller is the amplitude of the wave, the larger are the propagation distances required to develop observable changes in the wave profiles. For the propagation distances in our experiments (tens of cm) and the above amplitudes (few percent of the pump), waves display a linear behaviour.

For higher input amplitudes (50% of the pump) the wave distorts and self-steepens (red curve). As expected, the self-steepening observed in the experiments (Fig. 2(b,c)) is less severe than that observed in the simulations in the case of local media (e.g. in Fig. 1(b)), while is qualitatively similar to that shown in (Fig. 2(c), where the nonlocal medium response has been included. This clearly show how self-steepening is affected by a nonlocal nonlinearity, which has the effect of smoothening out any sharp features in the nonlinear response. Numerical simulations with the initial condition used in Fig. 1 but including a nonlocal response with the same *σ*, indeed confirm this interpretation [thin black line in Fig. 1(c)].

### Geometrical description

The connection between the nonlinear fluid equations and a “dynamic” acoustic metric can be shown by writing Eqs (3, 4, 5) in terms of the Riemann invariants, *ψ*_±_ = *v* ± 2*c*_*s*_, where *c*_*s*_ is the local speed of sound usually defined as 
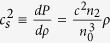
. In terms of these variables we obtain two advection equations


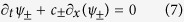


where 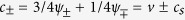. Eq. (7) simply state that the two variables *ψ*_±_ are conserved along the characteristics curves defined by *dx*/*dt* ≡ *c*_±_. Therefore, in a nonlinear wave the constant quantities *ψ*_±_ are propagated in spacetime at the characteristic speeds *c*_±_ = *v* ± *c*_*s*_, so they travel at the speed of sound relative to the flowing fluid. As in linear acoustic analogues, the characteristic curves demarcate the region of causally connected events. The acoustic line element can be written as


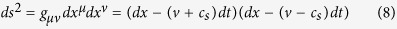


and thus the characteristics of the fluid equations coincide with the null geodesic defined by the acoustic metric *g*_*μν*_, whose coefficients depends on the fields *ψ*_±_.

The solutions *ψ*_±_ implicitly define the sound cone and so generate the above metric. However, depending on the initial consitions, they may generate other background structures. This is a difference with respect to GR, where spacetime metrics are the only background structures possessed by the theory and necessary to describe the dynamics. Other trivial solutions of Eq. (7) are: i) *ψ*_±_ = *const*, which corresponds to a fluid with constant density and constant flow. This would obviously modify the wave dynamics, while remaining globally unaffected waves propagating on it; ii) waves propagating in a single direction. These particular solutions fails to provide a metric, since they would define an incomplete sound cone. We could just define a single set of trajectories, the positive or the negative characteristics, by tracing the path in space-time of a hypothetical observer. In other words, we have not a causal structure that could be associated with a conformal class of Lorentzian metrics. However, for most of the initial conditions, Eq. (7) admit travelling wave solutions propagating in opposite directions, which thus generate a metric (8).

Notice that the advection equations (7) are weakly coupled through the coefficients *c*_±_ = *c*_±_(*ψ*_+_, *ψ*_−_), which depend on both Riemann invariants. Therefore, general wave solutions cannot be decomposed *exactly* into a sum of two *independent* left- and right-going waves as in the linear wave equation, where *c*_+_ = −*c*_−_ = *constant*. Nevertheless, it is possible to reconstruct an *approximate* solution which well describe the observed self-steepening dynamics up to the shock formation.

Consider an arbitrary density profile propagating on an uniform density state, *ρ*_0_, with zero flow (*v* = 0). After a short transient, the initial profile separates into two parts propagating in opposite directions, similarly to what observed in Fig. 1(b,c). When such right-going and left-going profiles are sufficiently well separated, they can be considered as nearly *independent*, in the sense that the right-going wave is not affecting the evolution of the left-going wave and viceversa. So, the complete solution is well approximated by the superposition of two components propagating in opposite directions. Such components, known as simple waves, can be thus independently calculated. Simple waves are particular solutions of Eq. 7 obtained by setting one of the Riemann invariants equal to a constant^32^. The known Riemann invariant allows us to eliminate one of the advection equations and thus to write the other in just one physical variable, either *ρ* or *v*. Consider the particular solution, *v*(*ρ*) = 2(*c*_*s*_(*ρ*) − *c*_*s*_(*ρ*_0_)), i.e *ψ*_−_ = *const*. The corresponding advection equation is identically solved and that for *ψ*_+_ can be written as ∂_*t*_*ρ* + (3(*c*_*s*_(*ρ*) − 2*c*_*s*_(*ρ*_0_))∂_*x*_*ρ* = 0. This equation describes a nonlinear wave propagating with velocity *c*_+_ = (3(*c*_*s*_(*ρ*) − 2*c*_*s*_(*ρ*_0_)). Similarly, we can now consider *v*(*ρ*) = −2(*c*_*s*_(*ρ*) − *c*_*s*_(*ρ*_0_)), i.e. *ψ*_+_ = *const*. and obtain the nonlinear advection equation for a wave propagating in the opposite direction with velocity *c*_−_ = −(3(*c*_*s*_(*ρ*) − 2*c*_*s*_(*ρ*_0_)).

The complete solution can be reconstructed via linear superposition of these two elementary components. We remark that such reconstruction cannot provide an exact solution of the nonlinear problem. On the other hand, when the wavepackets are sufficiently well separated their mutual coupling is negligible and then the solution is very well approximated by the superposition of the two independent simple waves calculated above.

Notice that any point in the wave profile (i.e. a point at a given density) propagates with constant velocity *c*_±_(*ρ*) = *v* ± *c*_*s*_, so the characteristics are now straight lines. Such waves can be conveniently regarded as a superposition of a density fluctuation propagating relative to the fluid, at the speed of sound and the movement of the fluid (induced by the wave itself) with velocity *v*. Since 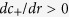 for right-going waves (and viceversa for left-going waves), the propagation velocity of a given point in the wave profile increases with the density: points of higher density propagate faster than points of lower density leading to self-steepening of the wave front. In a finite time the wave front will become vertical, implying an unphysical multivalued solution. The time and position in which the profile will exhibit such discontinuity is determined by the condition that *ρ*(*x*, *t*) has an inflection point with a vertical tangent line, i.e.


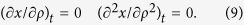


The latter conditions have a geometrical interpretation in terms of the characterisitics/null geodesics. Since the velocities *v* ± *c*_*s*_ are functions of the density, two characteristics of the same family (right-going or left-going) emanating from two spacetime points are not parallel and intersect at a finite time. As the gradient of the solution profile becomes increasingly steep, two characteristics become closer and closer. At the point of intersection, the solution is multivalued and the gradients are infinite (gradient catastophe)^32^.

The resulting spacetime is not null complete. In fact, the family of half null geodesics, (i.e. geodesic curves which have one endpoint and have been extended as far as possible from that endpoint), having a finite proper length, can be parametrized by a finite affine parameter^35^. Consequently, propagating waves reach a singularity in a finite time.

The appearence of a singularity can be explicitly shown by direct calculation of the Ricci scalar *R*, which in two dimensions fully determines the spacetime curvature. Considering the superposition of the two independent simple waves calculated above, we have *c*_+_ = (3(*c*_*s*_(*ρ*) − 2*c*_*s*_(*ρ*_0_)) = −*c*_−_. Substituting into Eq. (8), the metric takes the diagonal form


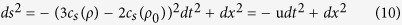


In two dimensions the Ricci scalar is twice the Gaussian curvature 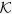, which for the above diagonal metric is given by


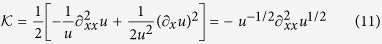


By using *u*^1/2^ = ±(3(*c*_*s*_(*ρ*) − 2*c*_*s*_(*ρ*_0_)) and *c*_*s*_(*ρ*) ∝ *ρ*^1/2^ we get


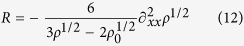


which, accordingly to Eq. (9), diverges at the shock formation point.

The fact that a multi-evaluated density (or flow velocity) would imply the divergence of *R* can be deduced from the definition of the acoustic metric, as already introduced in linear acoustic analogues. However, in linear models a singular spacetime can only be externally imposed, e.g by choosing a background with a topological defect. Here instead, a curvature singularity emerges spontaneously in a finite time, starting from a constant, homogeneous background. This is due to the *nonlinear* interplay between the propagating wave and the underlying metric, whose curvature is generated by the wave itself. Moreover, it is worth noting that since *ρ*_0_ = *const*., *R* has the same spatial dependence of the “quantum pressure” term in Eq. (4), 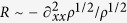. This is an interesting point that warrants further investigation. In linear analogue models, the quantum pressure is usually neglected in the derivation of the acoustic metric and, when is taken into account, is responsible for the high-energy breaking of Lorentz invariance. In a similar fashion, our geometrical analoguey has been established in the ideal pure nonlinear system (quantum pressure neglected). As a result, the nonlinear interaction (self-steepening) is the manifestation of the curvature of a dynamic spacetime metric generated by the wave. As time evolves, the wave (density) profile changes, thus modifying the curvature of the corresponding metric which, in turn, will affect the density profile. While at the initial stages of propagation the curvature/steepening is indeed negligible, it will forcibly increase in time and eventually will become infinite (singularity). It is known that in the presence of quantum pressure the singularity will never form: when the curvature becomes sufficiently strong, the quantum pressure comes into play and will eventually counterbalance the curvature. In this context, the fact that *R* has the same spatial and temporal dependence as the quantum pressure is not at all a coincidence. The perfect spatiotemporal matching between curvature (nonlinearity) and dispersive term (quantum pressure) is the unavoidable condition to have the compensation between the two effects, necessary to prevent the formation of the singularity. The resulting shock waves can be thus seen as spacetime structures of maximal –though finite– curvature.

**Figure 3:**
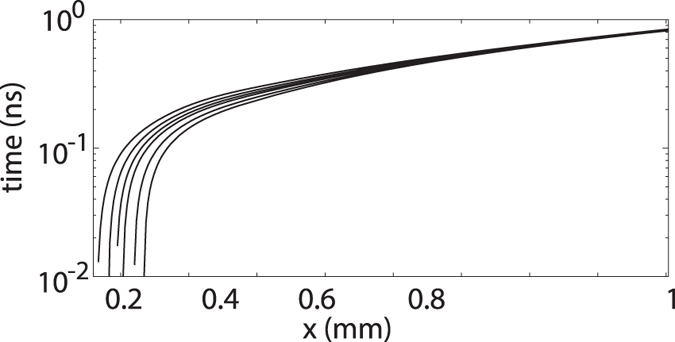
Numerical results. Trajectories of points with the same density generated by the right-moving high amplitude density wave shown in Fig. 1(c).

In Fig. 3 we plot the numerically calculated characteristics, corresponding to the (*x*, 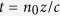) trajectories of points with the same density *ρ*, for the case of the nonlinearly propagating acoustic wave of Fig. 1(c). Although the presence of quantum pressure and nonlocal effects prevent the formation of a singularity, the convergence of the characteristics is a clear indication of an increasing spacetime curvature, in agreement with the ideal picture based on the emergent metric.

## Discussion

Quantum fluids such as BECs, polariton fluids and photon fluids have been proposed as platforms to study analogue gravity effects. To date these have focused on the propagation of weak amplitude density waves on top of a given background configuration. This has led to a series of important kinematic studies, including e.g. glimpses of Hawking radiation^36^. It is possible to extend these experimental models into the nonlinear regime where the background curved geometry determining the propagation of the waves is generated by the waves themselves. This self-interaction can thus be interpreted as kind of gravitational influence on the wave by its own effective metric. Such analogue nonlinear models are truer in spirit to general relativity, where mass distributions evolve in a spacetime metric that is modified by mass itself. However, as previously discussed, our backreaction is encoded in a set of nonlinear equations which are manifestly different from Einstein’s equations. In spite of this fact, in the presence of particular symmetries there is a precise correspondence between the gravitational field equations and the fluid dynamics. Therefore, suitably constrained photon-flows could be exploited to mimic the dynamics of gravitation black holes, spacetime singularities included^22^, and cosmological solutions^37^,^38^.

## Methods

Here we briefly describe the technique to estimate the relevant system parameters, *σ* and *ξ* (notice that the sound speed, *c*_*s*_, and the healing length, *ξ* are not independent parameters: *c*_*s*_*ξ* = *πc*/*kn*_0_. The characterization measurements are reported in ref. 23.

As the beam propagates through the nonlinear medium, the phase velocity of the sound wave is determined by *v*_*ph*_ = Ω/*K* with Ω given by Equation (6) and hence is a function of the system parameters. For low pump power, the nonlinear thermo-optic effect is negligible and the medium behaves essentially as linear optical system (*c*_*s*_ ≈ 0). In this limit, using Eq. (6) and the relation 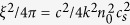, we calculate the phase velocity *v*_*ph*0_ = (*c*/2*kn*_0_)*K*.

As the pump beam power is increased we therefore expect to observe a spatial shift of the sound wave pattern by Δ*S* due to the increase of the phase velocity. The shift is given by Δ*S* = (*v*_*ph*_ − *v*_*ph*0_)*Ln*_0_/*c*, where *L* is the length of the medium, so the distance along which the wave has propagated. By using Eq. (6) and the above expression for Δ*S* we obtain


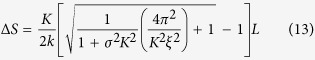


By measuring Δ*S* for different values of the pump power and for different wavevectors of the interference pattern, *K*, we may therefore use Eq. (13) to fit the data and estimate both *σ* and *ξ*. As a function of the wavevector *K*, one can recognize the finite limit Δ*S* → *π*/*kξ* for *K* → 0. Therefore for long wavelengths, the shift is observed to saturate to this limit value. The local vs. non-local nature of the nonlinearity is instead visible for short wavelengths.

## Additional Information

**How to cite this article**: Marino, F. *et al.* Emergent geometries and nonlinear-wave dynamics in photon fluids. *Sci. Rep.* **6**, 23282; doi: 10.1038/srep23282 (2016).
